# Microstructural Origins of the Corrosion Resistance of a Mg-Y-Nd-Zr Alloy Processed by Powder Bed Fusion – Laser Beam

**DOI:** 10.3389/fbioe.2022.917812

**Published:** 2022-07-01

**Authors:** Hanna Nilsson Åhman, Francesco D’Elia, Pelle Mellin, Cecilia Persson

**Affiliations:** ^1^ Division of Biomedical Engineering, Department of Materials Science and Engineering, Uppsala University, Uppsala, Sweden; ^2^ Swerim AB, Stockholm, Sweden

**Keywords:** hot isostatic press (HIP), additive manufacturing, powder bed fusion, laser beam, WE43, magnesium, biodegradable metals, biodegradation

## Abstract

Magnesium alloys are biocompatible, biodegradable and have the ability to promote bone ingrowth, making them ideal candidate materials for replacing auto- and allografts in future treatments of large bone defects. Powder bed fusion–laser beam (PBF-LB) additive manufacturing of these alloys would further allow for the production of complex structures, optimized for bone grafting. However, the corrosion rates of structures processed by PBF-LB remain too high. An improved understanding of the influence of the microstructure generated during PBF-LB on the corrosion properties is considered key to their future implementation in implants. In this study, the effect of PBF-LB processing and subsequent hot isostatic pressing (HIP) on the microstructure and texture in different sample directions was studied and related to the corrosion behavior of a Mg-Y-Nd-Zr alloy. The results were compared with an extruded Mg-Y-Nd-Zr alloy. A higher amount of secondary phases resulted in a higher rate of localized corrosion for the PBF-LB processed material compared to that for the extruded one. Due to growth of the secondary phases, the corrosion rate was further increased after HIP. Moreover, a strong texture was observed in the PBF-LB material, and it was also enhanced in the HIP material. While this affected the electrochemical activity as measured by potentiodynamic polarization tests, any texture effect appeared to be masked by the contribution of the secondary phases in the longer-term mass change and hydrogen evolution tests. Future work should look further into the influence of individual process parameters on the microstructure and the resulting corrosion behavior of the material, to further clarify its interdependence.

## Introduction

Bone tissue generally has a great ability to regenerate, and in the case of small fractures, it can heal without any outer intervention besides external support in the form of a cast. However, for the healing of large bone defects, such as comminuted long bone fractures, or defects related to the removal of diseased bone, surgical intervention is often needed (Einhorn and Gerstenfeld, 2015). Today, the gold standard for the treatment of large bone defects is autografting or allografting, where the missing bone is replaced with a piece of bone taken from another location of the patient’s own body or from a donor. Nevertheless, the limited availability along with the risk of donor site morbidity or host rejection remains a problem (Giannoudis et al., 2005; Valtanen et al., 2021). Consequently, alternative treatments for the improved healing of large bone defects are needed.

Magnesium (Mg) and its alloys have been found to be some of the most promising materials for the production of biodegradable orthopedic implants (Plaaß et al., 2016; Han et al., 2019; Prasad et al., 2021). Magnesium is highly biocompatible, biodegradable, and known to promote osteogenic differentiation and enhance bone ingrowth (Castellani et al., 2011; Cao et al., 2020; Liang et al., 2021). Furthermore, Youngs’s modulus of Mg alloys is closer to that of bone in comparison with the metal alloys traditionally used in orthopedics, such as the Ti-6Al-4V (Staiger et al., 2006; Kurdi et al., 2020). A mismatch in Young’s modulus will affect the distribution of stresses in the bone surrounding the implant. This is important, as bone is a dynamic material, and an incorrect stress distribution can result in bone degradation and implant loosening (Engh et al., 1987; Hasegawa et al., 2021). The first clinically implemented biodegradable metal implant was a bone fixation device produced from a powder extruded Mg-Y-Nd-Zr alloy, which received its CE certification in 2013 (Syntellix., 2019; Han et al., 2019).

The establishment of the Mg-Y-Nd-Zr family of alloys (e.g., WE43) has enabled a broader use of Mg alloys in a large range of industrial applications (Luxfer MEL Aircraft interiors., 2003; Kainer, 2003). Such Mg-RE alloys often consist in low alloyed (Mg > 90 wt%), multi-phase systems (Kainer, 2003) given their high tendency to form intermetallic compounds with Mg. The improved corrosion property of the Mg-RE alloys in comparison with other commercial alloys such as the Mg-Al-Zn systems (AZ-series), can be derived from the corrosion potential of the intermetallic phases formed in the Mg-RE system being closer to that of the Mg-matrix (Südholz et al., 2011). There is also a positive shift in the corrosion potential of the Mg–matrix due to the relatively high solubility of Y in Mg (Ben-hamu et al., 2007; Südholz et al., 2011). Moreover, Y contributes to an enhanced corrosion resistance by improving the stability of the surface through the formation of Y2O3 (Du et al., 2011). New production routes such as powder extrusion, ensure a uniform distribution of secondary phases to further promote a more uniform and less severe degradation rate, which is desired for resorbable orthopedic fixation devices and stents (Biotronik; Syntellix). (Magnesium Scaffold, 2020)

Powder bed fusion–laser beam (PBF-LB) is an advanced manufacturing technique offering unique possibilities regarding design optimization for all types of applications (Gibson et al., 2015). In orthopedics, PBF-LB of metal alloys such as Ti-6Al-4V has allowed for the manufacturing of complex patient specific implants with optimized structures for cell viability and bone ingrowth (Revilla-León et al., 2020; McGregor et al., 2021; Yang et al., 2021; Zhao et al., 2021). Combining the unique possibilities provided by PBF-LB regarding design optimization, with the biodegradability and biocompatibility of Mg alloys, can lead to the development of a new generation of load-bearing biodegradable metal implants for the improved healing of large bone defects. However, for a successful development of Mg-based biodegradable bone grafts, the corrosion rate needs to be controlled. Firstly, the corrosion rate needs to be slow enough, so that the biodegradable implant maintains its mechanical properties and structural integrity during the load bearing phase. For this, a degradation rate of 0.5 mm/year is the current recommended limit (Ding, 2016). Secondly, Mg alloys degrade with H2 gas as one of the main corrosion products. The amount of H2 gas formed needs to be controlled, as too high gas evolution can have an adverse effect on the bone remodeling (Plaaß et al., 2016; Meier and Panzica, 2017). A limit of an average H2 gas evolution rate of 0.01 ml/cm^2^/day has been suggested by Song et al. (Song et al., 2001).

The feasibility of processing Mg alloys by PBF-LB was first proven by Ng et al., in 2010 (Ng et al., 2010), while the first studies on the processing of a Mg-Y-Nd-Zr alloy specifically by PBF-LB were presented by Tandon et al. and Jauer et al., in 2015 and 2016, respectively (Tandon et al., 2015, 2016; Jauer et al., 2016). In the last couple of years there have been a number of studies on the microstructure and material properties of Mg-Y-Nd-Zr alloys processed by PBF-LB.

A few of these studies have focused on the corrosion properties of Mg-Y-Nd-Zr alloys processed by PBF-LB, including the effects of heat treatment (Esmaily et al., 2020) plasma electrolytic oxidation (PEO) surface treatments (Li et al., 2021), and lattice design (Kopp et al., 2019). A study on the *in vivo* degradation behavior of PBF-LB processed Mg-Y-Nd-Zr alloy has also been recently published (Liu J. et al., 2022). Esmaily et al. (Esmaily et al., 2020) evaluated the corrosion properties in 0.5% NaCl of a Mg-Y-Nd-Zr alloy processed by different PBF-LB parameters and subsequently treated by hot isostatic pressing (HIP) and heat treated. HIP treatment is commonly applied to remove defects (e.g., porosity) often found in PBF-LB materials in order to obtain a fully dense material (Gibson et al., 2015). Such defects can have detrimental effects on both corrosion and mechanical properties (Geenen et al., 2017). Esmaily et al. showed that HIP and heat treatment improved the short-term degradation rates, but the cast counterpart remained superior. Even though the study offered an important insight into the corrosion behavior of a Mg-Y-Nd-Zr alloy processed by PBF-LB, the study is limited to only short-term corrosion properties. Moreover, the corrosive medium did not contain neither Ca nor P, which have an important influence on the degradation properties of Mg alloys (Rettig and Virtanen, 2009; Esmaily et al., 2017; Py et al., 2020). In the case of surface treatments, although PEO has demonstrated the ability to mitigate the corrosion to some extent, the corrosion rates of the bulk Mg material remain too high (Benn et al., 2021; Li et al., 2021; Suchý et al., 2021). Additionally, the *in vivo* study further corroborated the need for further improvements of corrosion properties (Liu J. et al., 2022).

Finally, Mg-Y-Nd-Zr alloys produced by various routes, including PBF-LB, are known to produce a very strong texture (Esmaily et al., 2020; Li et al., 2021). Texture has previously been shown to affect the corrosion behavior of Mg alloys due to a difference in electrochemical activity of the different crystallographic planes (Song and Xu, 2012; Sabbaghian et al., 2019). Texture has also induced an anisotropic corrosion behavior in other metals processed by PBF-LB, such as Al (Chen et al., 2018) and Ti (Dai et al., 2016), but its effect on corrosion has not yet been evaluated for any Mg alloys.

With the aim of improving the corrosion properties of a Mg-Y-Nd-Zr alloy processed by PBF-LB, the porosities were removed through HIP. The resulting densification as well as its impact on the microstructure and texture and resulting corrosion properties for 28 days were investigated. The distribution and type of the secondary phases present in the Mg-Y-Nd-Zr alloy were characterized before and after HIP, along with grain size distribution and texture. Moreover, the influence of the resulting microstructure and texture on the long-term corrosion properties, including corrosion morphology and corrosion rate, was determined. Finally, the results were compared with those for a powder extruded Mg-Y-Nd-Zr alloy, which is the material from which the commercially available implants are currently being produced.

## Materials and Methods

### Sample Preparation

A gas atomized Mg-Y3.9-Nd3.0-Zr0.49 (in wt%) alloy metal powder, with a fine particle size distribution (d10 = 25 μm, d50 = 42 µm and d90 = 67 µm), was purchased from NMD GmbH (Hemseen, Germany). The powder was processed by PBF-LB on an EOS M290 printer (EOS GmbH, Krailling, Germany). The process was carried out under argon atmosphere (O2 < 0.1%) to minimize oxidation during the process. A laminar flow of argon across the bed removed the evaporation products. A build plate of a commercial Mg-Al-Zn alloy (KG Fridman AB, Karlstad, Sweden) was used to ensure the weldability of the powder to the build plate. Previously optimized parameters (Nilsson Åhman et al., 2022), with a laser power of 200 W, scanning speed of 1111 mm/s, hatch distance of 0.1 mm and a layer thickness of 0.03 mm, were applied to produce rectangular samples with a measurement of 10*10*30 mm. The laser focus diameter is 90 µm. The scanning direction was rotated 67° between each layer to minimize the overlapping of scanning tracks between layers (Pakkanen, 2018), and no contouring was used.

To understand the impact of porosity, two samples were post processed by HIP, to remove the final porosity. During HIP, 520°C and 105 MPa of pure argon was applied for 3 h in a QIH9 HIP machine (Quintus, Västerås, Sweden) (Esmaily et al., 2020).

Commercially produced powder extruded Mg-Y3.9-Nd3.0-Zr0.49 (in wt%) alloy was purchased (Smiths, Biggleswade, United Kingdom) and used as reference, as it corresponds to the material used in the biodegradable metal implants found on the market today (Syntellix).

### Compositional and Microstructural Characterization

**FIGURE 1:**
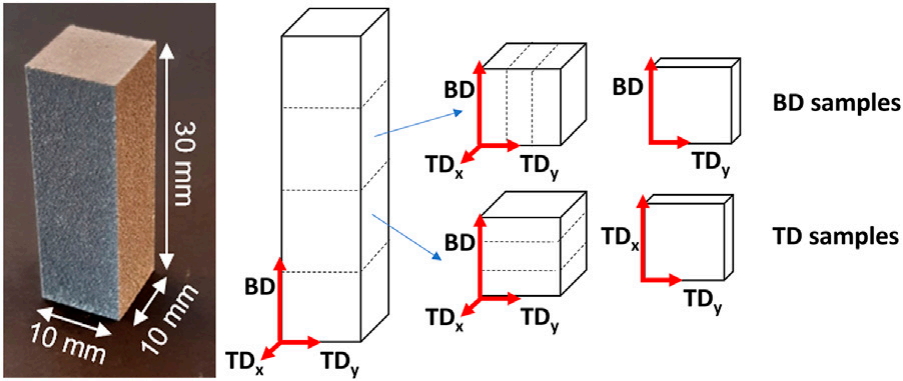
Schematic of the cutting of the samples indicating build direction (BD) and transverse directions (TD), as well as the denomination used for the surfaces throughout the text.

The porosity of the as-built (AB) and HIP samples was established according to the standard ASTM B311-17, using the Sartorius YDK01 density determination kit (Sartorius AG, Goettingen, Germany). The density of the samples was measured before they were cut according to the scheme presented in Figure 1, with NAB = 5 and NHIP = 2. The density was then compared to the expected density of the alloy, 1.84 g/cm^3^ (Cverna et al., 2001).

To study the microstructure of the surfaces in the build direction (BD) and the transverse direction (TD) the samples were cut according to the scheme presented in Figure 1 to obtain 10 mm^2^ surfaces. The BD surface is defined as the surface that is parallel to the build direction, i.e., the *z* axis, and perpendicular to the *x* and *y* axes. The TD surface is defined as being perpendicular to the *z* axis, and thus the BD surface. As the scanning direction was rotated with 67° in each layer, the *x* and *y* axes can be used interchangeably, i.e., TD = TDx and TDy.

For microstructural analysis, samples of the AB, HIP and extruded (Extr) material were mounted in Bakelite and ground with abrasive silicon carbide papers of grades P600, P1200, P2500 and P4000, and subsequently polished using oxidized porous silica (OPS) for 15 min. The microstructure of the samples was investigated using a light optical microscope (LOM) (Leica DM IRM, Leica Microsystems GmbH, Wetzlar, Germany), after 5 s of etching with 2% Nital (2 wt% nitric acid in methanol). Further characterization of the microstructure and the secondary phases was done by backscatter electron imaging (BSE) in a scanning electron microscope (SEM, AZtec 5.0, Oxford Instruments, Abingdon, United Kingdom).

Identification of secondary phases was also done using X-ray diffraction (XRD) (Bruker D8 Discover, Bruker, Billerica, MA, United States). The XRD was performed with Cu Kα (1.54 Å) radiation, and poly-capillary optics were used for parallel beam geometry with a collimator size of 2 mm. Data was collected between 10° and 120° (2θ) and analyzed using the Bruker software DIFFRAC.EVA (Bruker, Billerica, MA, United States).

Grain size and texture of the AB, HIP and Extr material were measured using electron backscatter diffraction imaging (EBSD, Nordlys HKL detector, Oxford Instruments, Abingdon, United Kingdom). A step size of 0.8 µm was used, and the grain boundaries were defined as having a misorientation greater than 15°. The grain size is defined as the area-weighted average of the equivalent circle diameter. The data was analyzed using Aztec Crystal 2.0 (Oxford Instruments, Abingdon, United Kingdom).

### Corrosion Measurements

For corrosion measurements, 1 mm of the AB surface of the 10*10*30 mm printed rectangle was removed to ensure that only bulk material was being evaluated. Samples were then cut into pieces measuring 8*8*2 mm according to the scheme presented in Figures.

Figure 1 to ensure that the surface investigated was the one mainly in contact with the corrosion medium. Samples of the same size were sectioned from the Extr material, investigating the surfaces in the extrusion direction. The surfaces were ground with abrasive silicon carbide papers of grades P600, P1200 and P2500, then washed with ethanol, dried with a hot air dryer, and stored in a desiccator for 24 h before evaluation. All corrosion tests were carried out in non deareated Dulbecco’s Phosphate Buffered Saline solution (DPBS, Sigma Aldrich, St. Louis, MO, United States) with the concentrations given in Table 1. The ratio between the corrosion medium and the sample surface area was above 20 ml/cm^2^, in accordance with ASTM G31 (ASTM G31-72 (2004))

**TABLE 1:** Salt concentration of Dulbecco’s Phosphate Buffered Saline solution.

	CaCl2 ∙H2O	MgCl2∙H2O	KCl	KH2PO4	NaCl	Na2HPO4
g/L	0.133	0.1	0.2	0.2	8.0	1.15

Potentiodynamic polarization testing (PDP) was performed in triplicate using a flushed-port cell with a three-electrode set-up (ASTM G150) (ASTM G150-18, 2018), and a Parstat 3000A-DX potentiostat (Ametek Inc., Berwyn, PA, United States). A saturated calomel electrode was used as a reference, and Pt-wire as a counter electrode. Potentiodynamic scans were conducted at a rate of 1 mV/s, after 10 min of conditioning at open circuit potential (OCP) (Kirkland et al., 2012). The corrosion potential (Ecorr) and corrosion current (icorr) were determined by Tafel extrapolation using VersaStudio software (VersaStudio 2.60.6, Ametek Inc., Berwyn, PA, United States).

To further investigate the initial stages of the corrosion attack in the AB, HIP and Extr material, samples were ground with abrasive silicon carbide papers down to P4000, and subsequently polished using OPS for 15 min, before immersion in DPBS for 30 min. The resulting surface was investigated with BSE - SEM.

To study the corrosion morphology and the corrosion behavior over time, immersion tests in triplicate were also carried out in DPBS at 37°C for 28 days. An analytical balance (Mettler AE240, Mettler-Toledo, Columbus, OH, United States) with an accuracy of 0.05 mg was used to establish the weight of the samples before and after 28 days of immersion. To track the instantaneous corrosion rate, the hydrogen evolution was also measured over a period of 28 days using the volumetric method described by Song et al. (Song et al., 2001). The samples that were immersed for 28 days were washed with deionized water and dried with a hot air dryer, and their surfaces were then examined in LOM to establish the corrosion morphology.

Finally, the samples immersed for 28 days were mounted in Bakelite, and ground with abrasive silicon carbide papers of grades P600, P1200, P2500 and P4000, using ethanol, and subsequently polished with diamond carbon paste with grit size 3 and 0.25 µm. The resulting cross sections were characterized using BSE-SEM and EDS.

## Results and Discussion

### Compositional and Microstructural Characterization

**FIGURE 2:**
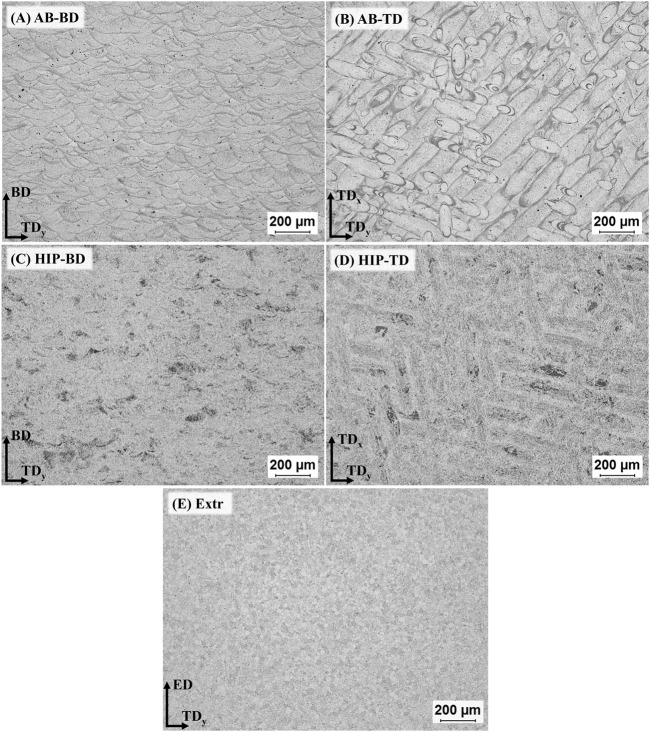
LOM images of the samples after 5s etching in 2% Nital, for **(A)** AB-BD direction, **(B)** AB-TD direction, **(C)** HIP-BD direction, **(D)** HIP-TD and **(E)** Extr material.

The result for the densities of the AB and HIP samples was 99.4 ± 0.05% and 99.9 ± 0.005% respectively. According to the Archimedes measurement there is a clear increase in density after HIP, and no pores are visible in the microscopy images of the HIP material (Figure 2). The LOM images of the AB and the HIP samples, both in BD and TD direction, as well as the Extr sample are presented in Figure 2. The individual melt pools are clearly visible in the LOM images of the AB sample in the BD direction (Figure 2A)), and conversely, the scanning lines from the laser are clearly visible for AB sample in the TD image (Figure 2B)). Examining the LOM images after HIP, the structure of the melt pools is still discernable in the images of the HIP material in the BD direction (Figure 2C)) and the scanning lines from the laser are still clearly visible in the HIP material in the TD direction (Figure 2D)). As there is usually a preferential etching taking place in areas surrounding secondary phases, one can from these images presume that there is a redistribution of secondary phases after HIP. In the Extr material, there is a segregation of secondary phases along the grain boundaries as well as typical necklace-type distribution of particles, and the individual recrystallized ⍺-Mg grains are clearly visible (Figure 2E)).

**FIGURE 3:**
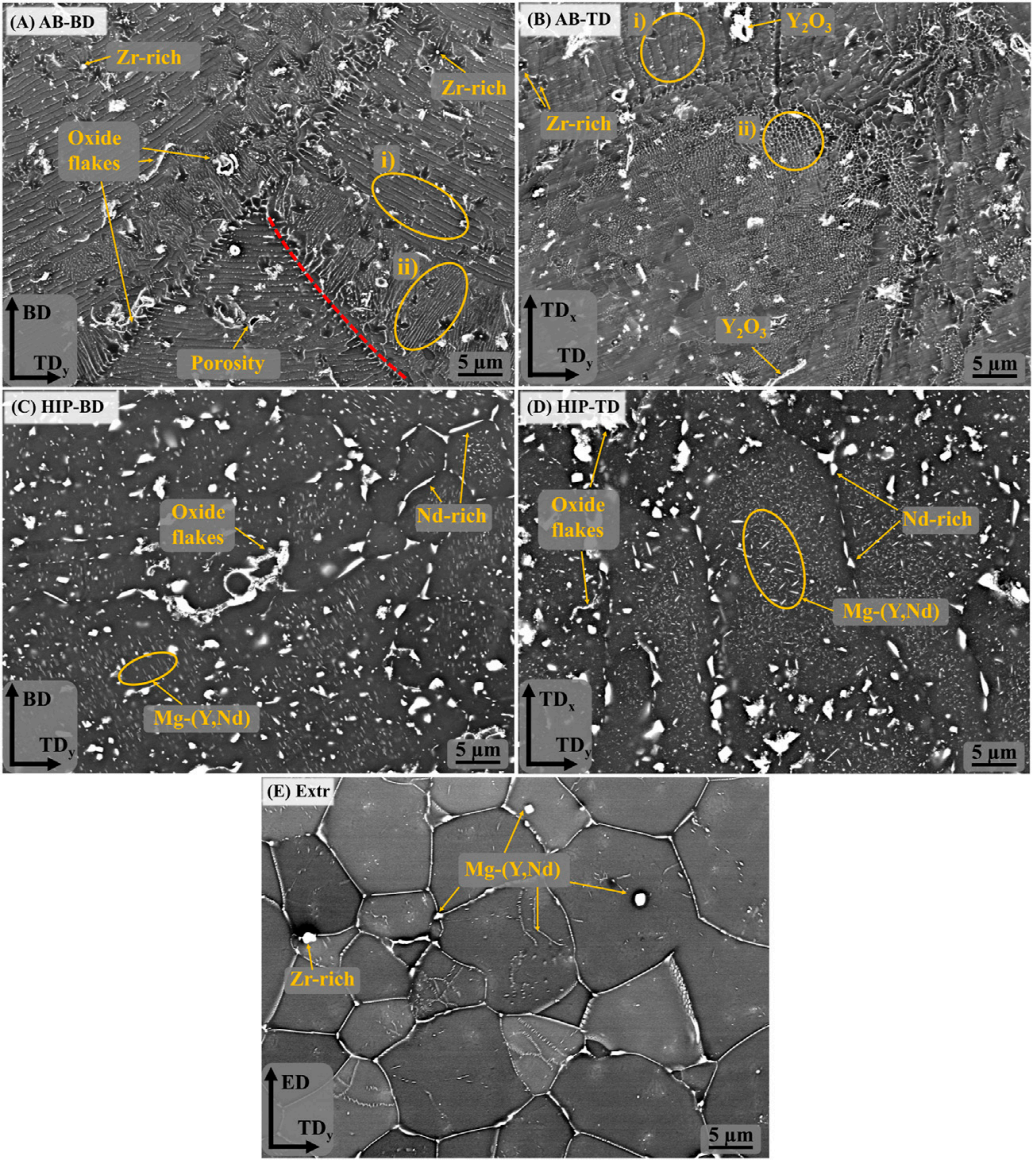
BSE-SEM images of the **(A)** AB-BD direction, **(B)** AB-TD direction, **(C)** HIP-BD direction, **(D)** HIP-TD and **(E)** Extr material.

The melt pools are also clearly visible in the BSE-SEM images, marked with a dashed red line in Figure 3A). In the same image, the area in the circle marked i) highlights a cellular structure that can be seen inside the melt pools, with lines of precipitates aligned parallel to the melt pool boundaries. The size of the precipitates ranges from a few nm up to 100 nm. At the melt pool boundaries is another region, highlighted by the circle marked ii), where precipitates are organized in lines perpendicular to the melt pool boundary. These regions coincide with the area marked with i) and ii) in the BSE-SEM images of the TD direction (Figure 3B), confirming the accumulation of secondary phases at the edges of the laser scanning track. Moreover, round precipitates with sizes around 200–400 nm are present throughout the material independently of the melt pool boundaries, as indicated in Figure 3A. EDS point analysis identified them as Zr-rich particles. Larger precipitates in the form of flakes measuring a few micrometers and high in Y and O are also visible throughout the microstructure. These oxide flakes are likely remains from oxide shells originating along the surface of powder particles during gas atomization. Due to the very high melting point of these oxide shells, it is unlikely that they are melted during PBF-LB, but rather, they are cracked and therefore remains inside the solidified material. Precipitates of HCP-Zr have been observed before, and have been predicted by thermodynamic calculations based on the Calphad method (Nilsson Åhman et al., 2022). These observations are in line with what has previously been observed for a Mg-Y-Nd-Zr processed by PBF-LB (Bär et al., 2019).

After HIP, the secondary phases coarsen considerably, and the melt pool boundaries are no longer visible in BSE-SEM images (Figures 3C,D). There are randomly distributed larger angular precipitates in the order of µm, rich in Nd and there are also smaller precipitates in the form of thin platelets and globular particles, ranging in size from a few to 100 nm. EDS found the area containing these precipitates to be enriched in Y and Nd. The oxides are still present after HIP, which was expected, as the solubility of O in Mg is very low (Hallstedt, 1993). Inspection of the BSE-SEM images of the Extr material reveals secondary phases rich in Y and Nd that are primarily present in the grain boundaries, whose distribution corresponds to that also observed in the LOM images in Figure 2. Some Zr-rich precipitates are also present, but none were found to contain large amounts of O.

**FIGURE 4:**
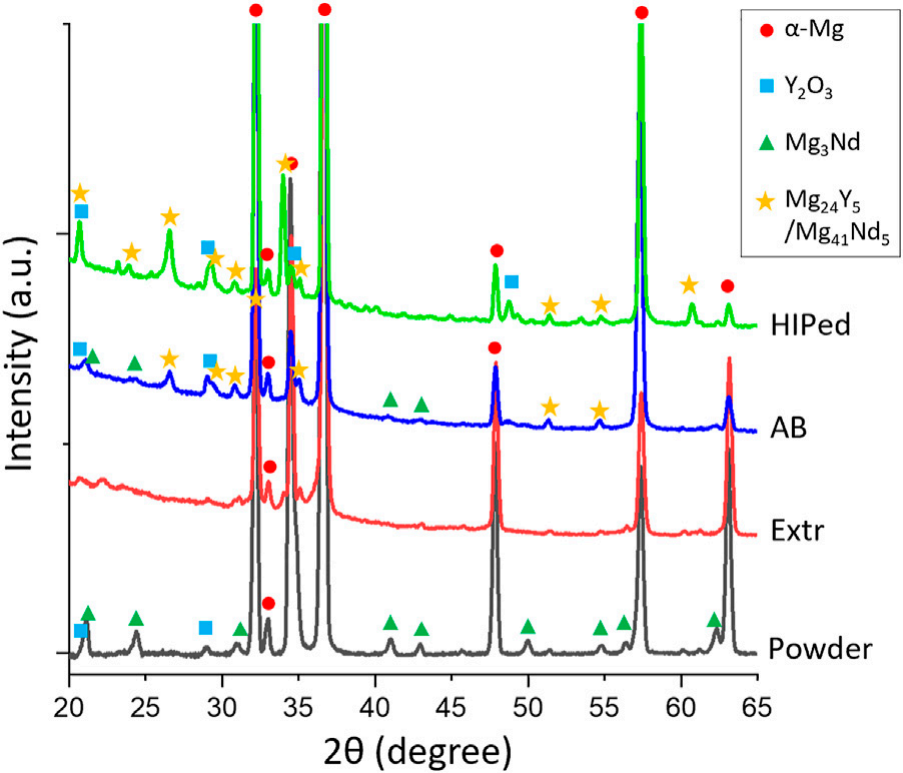
XRD spectra of the powder, the Extr, AB and HIP samples.

The XRD spectra for the AB, HIP, Extr and powder material is presented in Figure 4. The main peaks all correspond to α-Mg in all spectra, and smaller peaks identified with Y2O3 were also present in all samples. Oxide particles were observed in the microstructure of the Extr material, originating from the production of the powder, but is also expected to be found as a thin layer on the surface. In the powder and in the AB material, peaks corresponding to the Mg3Nd phase were established. However, no peaks corresponding to the Mg3Nd phase were seen for the HIP material. Peaks that are not present in the powder sample but can be found in the spectra for both the AB and the HIP material are those corresponding to the intermetallic compounds Mg41Nd5 or Mg24Y5. It is not possible to determine which is present due their peaks overlapping in the XRD spectra, but both are expected to be present, together with ternary Mg-Y-Nd phases (Zumdick et al., 2019). These peaks also grow stronger after HIP, which could be related to the growth in size of these secondary phases observed in the BSE-SEM images (Figure 3). There are also a number of smaller peaks that were not identified.

The LOM and BSE-SEM microscopy images as well as the results from the XRD are in line with the microstructure observed for the AB and Extr material in previous studies (Bär et al., 2019; Zumdick et al., 2019; Esmaily et al., 2020). Mg3Nd is a meta-stable intermetallic phase, and the presence of the Mg3Nd precipitates in the powder, as well as in the AB material, is also in agreement with the discussion on stability and thermodynamics of the secondary phases in Mg-Y-Nd-Zr alloys (Nie and Muddle, 2000). As it is a meta-stable phase, this can also explain why it is not present in the material after HIP. The intermetallic compounds Mg41Nd5 and Mg24Y5 have been confirmed as stable phases in a number of other studies on the Mg-Y-Nd systems, and are also the precipitates present in the HIP material (Gangireddy et al., 2018).

**FIGURE 5:**
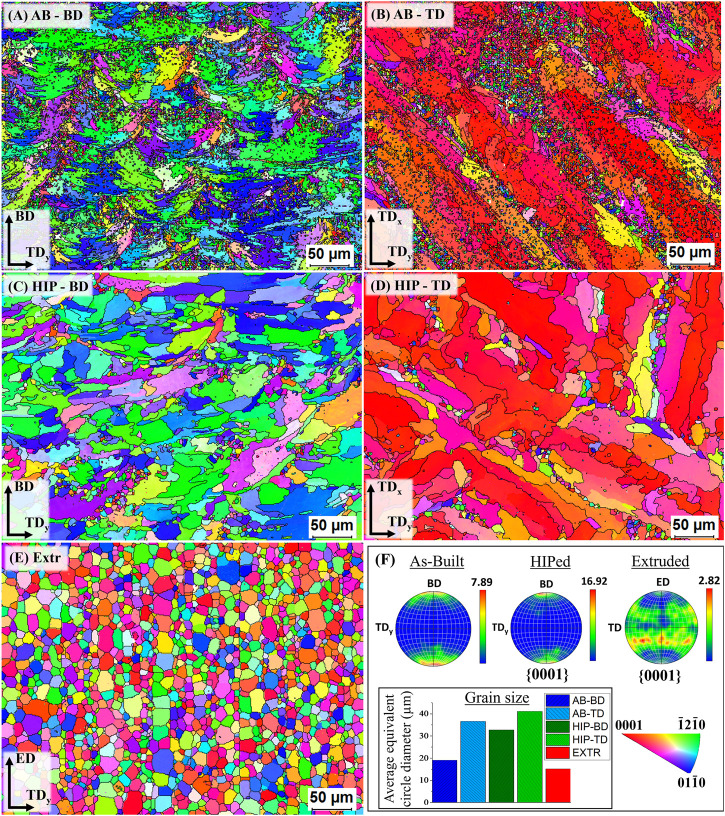
IPF color maps of the AB **(A**,**B)** HIP **(C**,**D)** and Extr **(E)** material, together with the pole figures **(F)**.

The result of the EBSD measurements of the AB, HIP and Extr material can be found in Figure 5 in the form of inverse pole figures (IPF) and corresponding IPF color maps. The IPF color maps of the AB material in the BD and TD direction can be seen in Figures 5A,B. In both these images it can be observed that grains have to a large extent grown within the melt pool. It is especially evident in the IPF color map of the AB TD image, where larger grains with a strong basal texture grown within the laser tracks can be observed. The extension of the larger grains along the melt pool results in a larger grain size in the TD direction than in the BD direction. The average grain size in the BD direction was around 19 μm, while for the TD direction it was around 35 µm. The larger grains have a crystal structure whereby the [0001] axis of the HCP crystal grains is orientated parallel to the build direction. Along the boundaries of the laser scanning tracks, there are clusters of smaller equiaxed grains with random texture.

The two types of grain structures have been observed in previous work, with a varying distribution. Jauer et al. reported an average grain size of around 1 µm in the BD direction (Jauer et al., 2016), with the majority of the grains having equiaxed morphology and weak texture, and only a few large elongated grains having basal texture. The information given on the process parameters is however too limited to discuss the difference. Esmaily et al. on the other hand, reported average grain sizes (Esmaily et al., 2020) ranging from 18 to 36 µm in the TD depending on the processing parameters applied. The majority of the grains consisted in the larger grains with a strong basal structure, and only a limited amount of the smaller equiaxed grains with a weaker texture can be observed in the EBSD map for one of the sets of process parameters. Esmaily et al. also applied a different scan strategy, rotating 90° between the layers, as opposed to the 67° rotation used in this study. The difference in scanning rotation has resulted in a difference in grain growth in other metals processed by PBF-LB, such as in Ti alloys (Zheng et al., 2022) and in Ni alloys (Carter et al., 2014). However, no major difference was observed in the morphology of the larger grains in this study and the article by Esmaily et al. However, there is a large number of the smaller equiaxed grains present in this study. This could be related to the difference in hatch distance in relation to the focus diameter of the laser beam. Esmaily et al. used a hatch distance of 40 μm, which is much smaller than the focus diameter of 90 µm. Comparing these parameters to those used in this study, i.e., hatch distance of 100 µm and focus diameter of 90 μm, suggests that subsequent scanning lines in the study of Esmaily et al. were overlapping to a much larger extent. This larger overlapping of subsequent laser scanning tracks would result in increased remelting of the material and thus, a lower presence of smaller equiaxed grains. Moreover, there are differences in laser power and laser scanning speed, thereby making it difficult to conclude on the extent to which the various parameters affect the difference in microstructure between the two studies.

Neither Esmaily et al. nor Jauer et al. investigated the difference in grain morphology between TD and BD. Zumdick et al. (Zumdick et al., 2019) obtained an average grain size of 1.0 ± 0.4 µm and 1.1 ± 0.4 µm for the TDx and TDy respectively, and 1.1 µm in the BD plane, which is much smaller than those observed in this study. However, the authors only reported average grain sizes and did not regard the effect of grain orientation and texture in their study. Until now, the differences in grain size distribution depending on the build direction, has not been investigated.

The differences observed in reported microstructures between previous studies could be due to variations in both the processing parameters, as well as the original alloy content of the gas atomized powder. However, these are the only studies available that include characterization of the grain size of Mg-Y-Nd-Zr alloys processed by PBF-LB. Regardless, the difference in grain morphology observed in the above-mentioned studies, as well as the clear difference between the TD and BD direction observed in this study, highlights the need to further study the relationship between feedstock, processing parameters and microstructure, as well as its influence on material properties.

The IPF color maps of the HIP material can also be seen in Figure 5. In these images it can be observed that the smaller equiaxed grains have almost disappeared, and the grains with strong basal structure have grown, resulting in an increase in average grain size in both the BD (33 µm) and TD (41 µm). It should also be noted that the average grain size for AB-TD is larger than for HIP-BD. As a result of the growth of the larger grains with a strong basal structure, and the equiaxed grains with a random texture disappearing, the HIP material exhibits a much stronger texture than the AB material. These results show a larger change in grain structure as compared to what has previously been reported for the HIP Mg-Y-Nd-Zr alloys processed by PBF-LB and HIP with same parameters (Esmaily et al., 2020). Esmaily et al. also observed a larger amount of equiaxed grains remaining after HIP.

In the IPF color map of the Extr material, the same grain structure as observed in the LOM and the BSE-SEM images can be seen. The Extr material has an average grain size of 15 μm, with more homogenous grain size distribution, and a weaker texture than that of the PBF-LB material. These results are in line with what has previously been reported for extruded Mg-Y-Nd-Zr alloys (Zhang et al., 2020).

**FIGURE 6:**
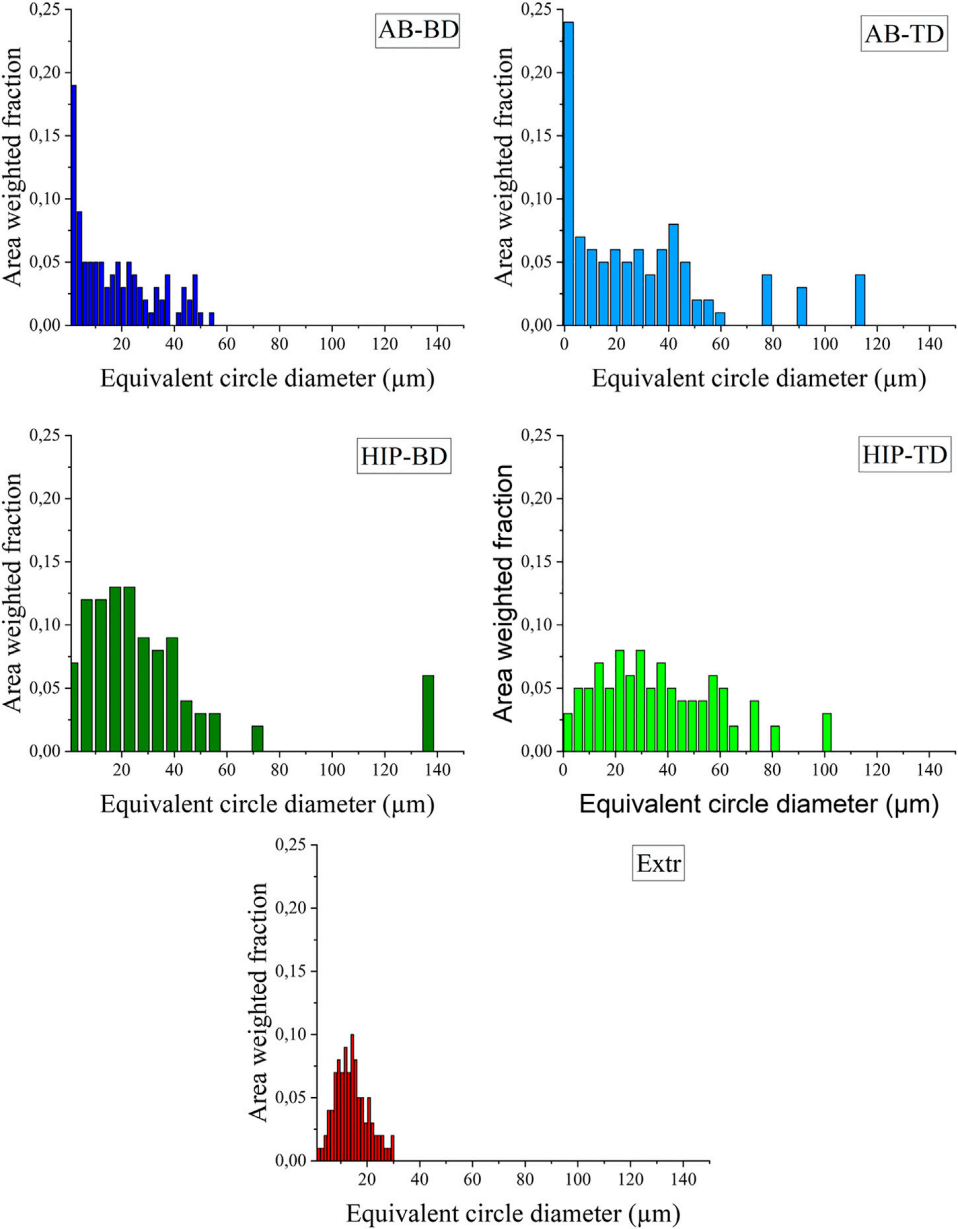
Grain size distribution presented as equivalent circle diameter plotted against the area weighted fraction for the **(A)** AB-BD, **(B)** AB-TD, **(C)** HIP-BD, **(D)** HIP - TD and **(E)** Extr material.

The observations made for the EBSD color maps presented in Figure 5 can be corroborated by the graphs presenting the grain size distribution (Figure 6). For both the AB groups, a larger number of small grains are present than for the HIP material. The increase in larger grains can also be seen for the HIP material. The higher amount of large grains in the TD material compared to the BD material can also be observed for both the AB and HIP material. The extruded material is, as can be expected from the EBSD map, showing a normal distribution.

### Corrosion Measurements

**FIGURE 7:**
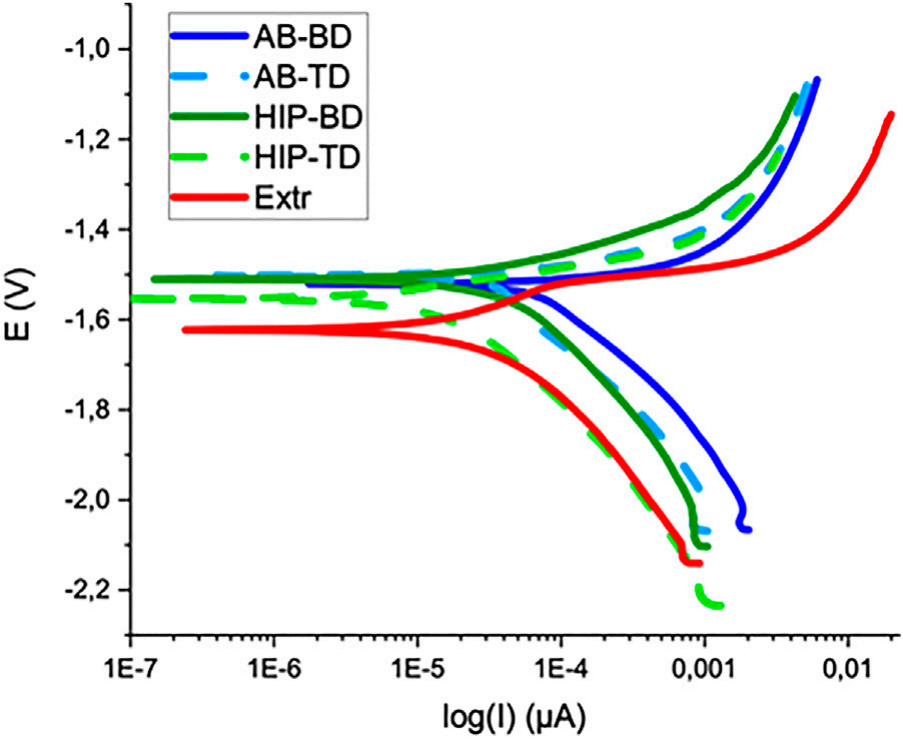
Potentiodynamic testing curves for AB, HIP and Extr material.

The corrosion properties of the samples were first evaluated by PDP, for which the results are presented in Figure 7, together with the averaged values of the open circuit potential (PCP) corrosion potential and the corrosion current in Table 2. In contrast to the printed material, the Extr material showed a slight tendency towards passivation, manifested as an inflection in the anodic branch of the curve, which could indicate an initial breakdown followed by a passivation of the surface. Neither the AB nor the HIP material showed a similar behavior. The BD and TD surfaces all show a similar anodic behavior, indicating high corrosion rates for all printed samples. However, at higher potentials the anodic current density was highest for the Extr material.

**TABLE 2:** OCP, corrosion potential, and corrosion current for respective material.

	OCP	Ecorr	Icorr
	[Vvs SCE]	[Vvs SCE]	[µA/cm2]
AB-BD	−1.56	−1.51 ± 0.006	119 ± 61.2
AB-TD	−1.58	−1.51 ± 0.007	37 ± 8.2
HIP-BD	−1.58	−1.49 ± 0.020	53 ± 15.9
HIP-TD	−1.60	−1.57 ± 0.04	25 ± 13.5
Extr	−1.65	−1.62 ± 0.002	30 ± 4.1

With regards to the cathodic branch of the curve, the AB materials show higher current densities than both the HIP and Extr material. The AB current densities are higher throughout the cathodic branch of the polarization curve than the HIP and this shift in the cathodic part of the curve is also reflected in the evaluated corrosion current densities (icorr), which are higher for AB than the corresponding HIP surface, indicating that the surface of the HIP material is more stable.

Comparing the current densities for the BD to the TD surfaces of the AB and HIP material, both BD surfaces exhibit a higher corrosion current than the corresponding TD surface. The difference was more pronounced for the AB than for the HIP material. Looking at the result obtained from EBSD, this is in line with the findings of Song et al. (Song and Xu, 2012) which showed that the strongly textured Mg-Al-Zn alloy exhibited a lower corrosion activity in the basal plane, as the close packed (0001) plane has a lower surface energy and is thus more stable and more corrosion resistant. Moreover, the variation in results for the printed samples were larger than for the Extr material, indicating a more inhomogeneous material.

**FIGURE 8:**
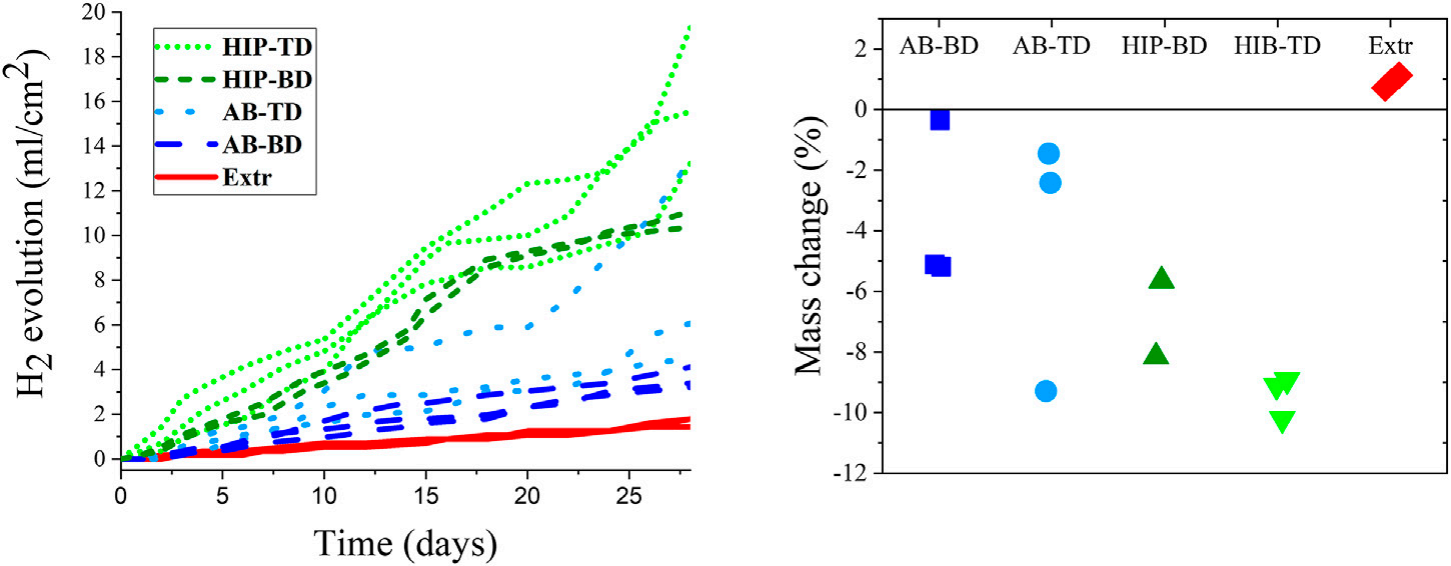
Hydrogen evolution measured over a period of 28 days **(A)** together with a scatterplot for the change in mass after 28 days of immersion **(B)**. (*n* = 3 for all sample groups but HIP-BD, where *n* = 2).

The results from the hydrogen evolution and the mass change are presented in Figure 8. Looking at the hydrogen evolution, it is clear that the HIP material exhibited the highest hydrogen evolution rate, followed by the AB material. The results for the TD and BD surfaces were overlapping. The powder Extr material exhibited the lowest rate of hydrogen evolution, and also had the lowest divergence between the samples. The higher divergence indicates a more inhomogeneous material, which is in line with the corrosion current densities previously established (Figure 7). These results contradict the results for the PDP, which indicated that the AB material had a higher corrosion rate than the HIP one. A reason for this contradiction could be that the porosity in the AB material results in a higher surface area being exposed and has greater importance during the short-term PDP testing. Furthermore, the PDP is an accelerated test, and the formation of protective corrosion layers at the surface could make an appreciable contribution to the measured current densities. The hydrogen evolution and mass change experiments are carried out for a much longer time and thus more closely reflect the expected performance as implants.

**FIGURE 9:**
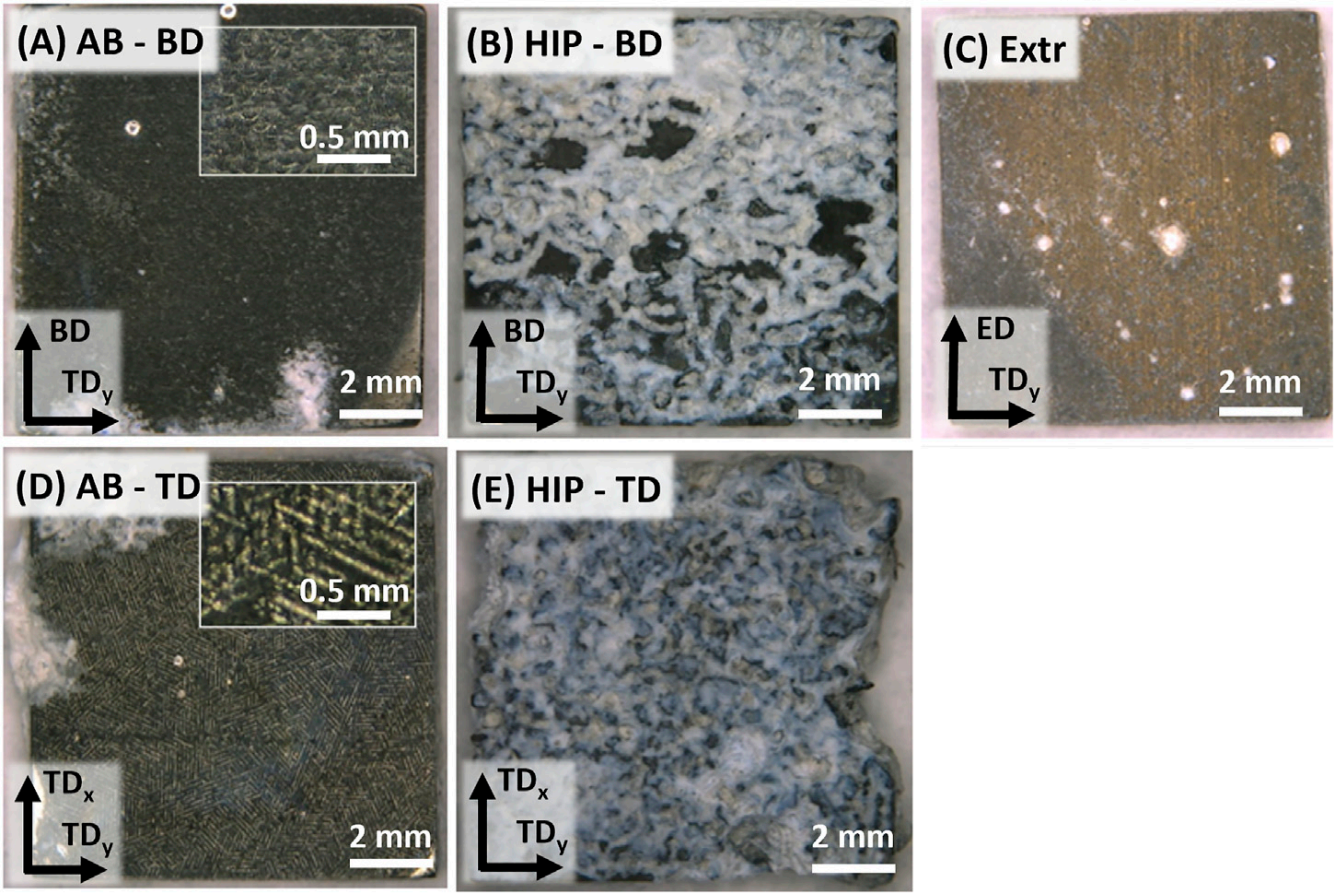
Images of corroded samples after 28 days of immersion, with the AB surfaces in **(A**,**D)**, the HIP surfaces in **(B**,**E)**, and **(C)** the Extr material.

The mass change is also presented in Figure 8 indicating similar results as the hydrogen evolution, with the HIP material having the highest corrosion rate followed by the AB material. The mass change results are affected by the buildup of corrosion products on the surface of the samples, which explains the positive mass change for the Extr material. There is also a large span in mass change, especially for the AB samples, which indicates an inhomogeneous material. Moreover, the number of samples available were limited, which limits drawing any strong conclusions regarding the difference BD and TD surfaces from the mass change measurements. Nevertheless, a clear difference between the samples could also be observed macroscopically in the LOM images of the samples after 28 days of immersion (Figure 9).

The images in Figure 9 show the same trend as for the hydrogen evolution and mass change results, with the HIP material exhibiting the most severe corrosion attack, followed by the AB, and finally the Extr material. A thick layer of insoluble white corrosion products was formed on the surfaces of the HIP material (Figure 9B), and there was a large amount of corrosion products piled up at the bottom of the beaker for these samples. Even though some areas of the AB material also exhibited a buildup of the white corrosion products, and a small amount was collected at the bottom of the beaker, the AB and the Extr material both maintained a more homogenous surface (Figures 9A,C). Inserts of higher magnification for the AB material in Figure 9A still reveal the structure resulting from the laser scanning tracks (as observed in the LOM and BSE-SEM images of the AB material), especially in the TD direction. While the surface of the AB material turned dark, the surface of Extr material surface maintained a brighter gold-like color throughout the 28 days. Mg alloys darken as a thicker layer of corrosion products form on the surface, hence the difference in color indicates that the Extr material had a thinner layer of corrosion products built up on the surface (Taheri et al., 2014; Esmaily et al., 2017).

**FIGURE 10:**
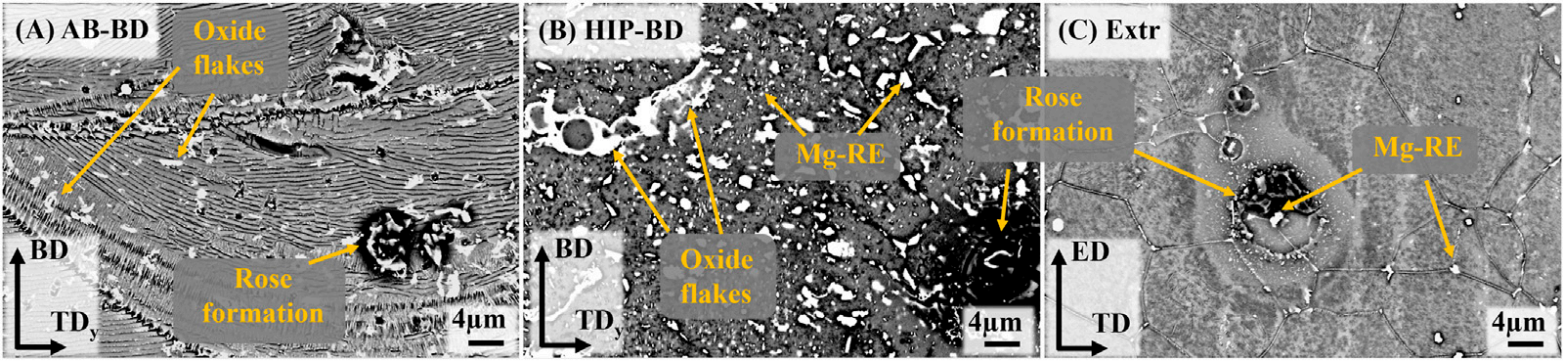
BSE-SEM mages of the surfaces of **(A)** AB, **(B)** HIP and **(C)** Extr samples after 30 min immersion in DPBS.

In order to achieve a better understanding of the corrosion mechanism at play, the surfaces of the samples were studied in the SEM after 30min immersion in DPBS (Figure 10). Local attack in the form of rose formations consisting in a buildup of corrosion products was observed in all materials. This type of formation has been previously observed to form around cathodically active intermetallic particles for both cast and Extr Mg-Y-Nd-Zr alloys (Kalb et al., 2012; Ascencio et al., 2015). This visibly local attack is in agreement with the behavior observed in the PDP curves, where the exponentially increasing anodic branch is indicating pitting corrosion (Kirkland et al., 2012). Although these rose formations are present also in the Extr material, the attack is less severe in other areas of the material, as can be seen in Figure 10C.

Comparing the Extr material (Figure 10C) with the AB material (Figure 10A), the corrosive attack, corresponding to the dark areas surrounding the intermetallic particles, is more severe in the AB material. Moreover, comparing the AB material to the HIP material, the dark areas surrounding the intermetallic particles are even larger for the HIP material. The difference between the printed materials and the Extr material can be related to the larger amount of intermetallic particles in the printed material as opposed to the extruded material, also resulting in a lower amount of alloying elements being dissolved in the Mg matrix. The difference between the AB and the HIP material was explained by the growth of the amount of intermetallic particles. It can also be observed that the localized attack is not as severe around the oxide flakes, suggesting that the internal oxides might not be the main cause for the poor corrosion resistance of the PBF-LB material, and that the Zr-rich and Mg-RE intermetallic particles are of greater relevance. However, even though the oxides do not appear to be a primary site of attack, a larger amount of Y2O3 would lead to a depletion of Y from the matrix, decreasing the corrosion resistance of the material (Davenport et al., 2007). Corrosion studies of Mg-Y-Nd-Zr alloys processed by PBF-LB and heat treated by other methods have exhibited similar results, with the heat-treated material generally possessing worse corrosion properties (Kopp et al., 2019; Li et al., 2021).

**FIGURE 11:**
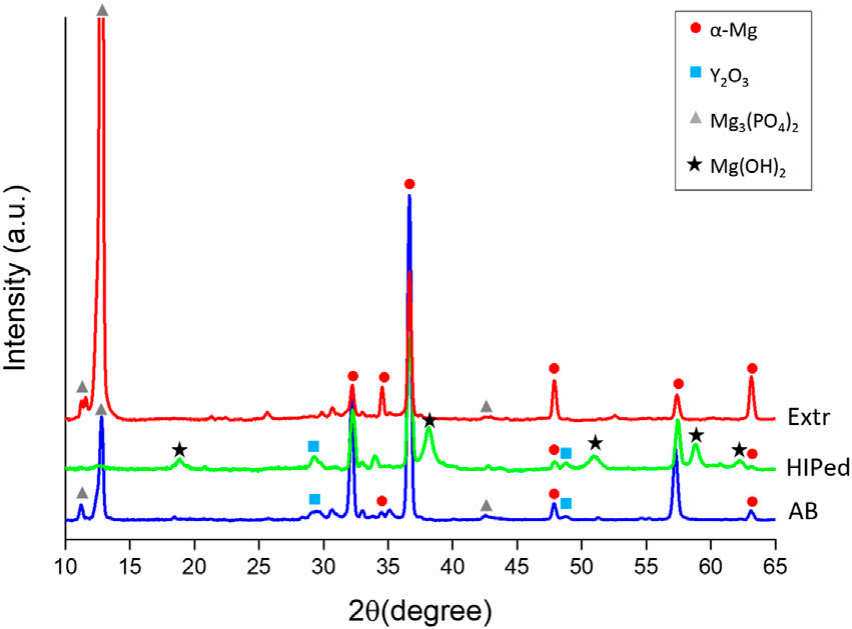
XRD of the BD surfaces of the AB and HIP samples, as well as the Extr samples after 28 days of immersion in DPBS.

**FIGURE 12:**
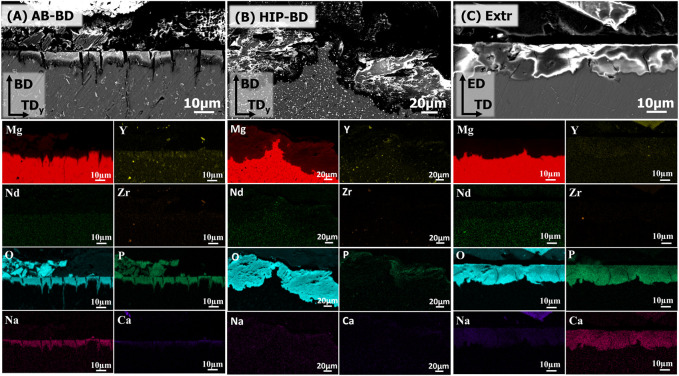
BSE-SEM images of the cross-sections of the corroded **(A)** AB, **(B)** HIP and **(C)** Extr samples, after 28 days of immersion. The EDS maps of the main alloying elements (Mg, Y, Nd, Zr) together with the main elements found in the corrosion products (O, P, Na, Ca) are included below corresponding BSE-SEM image.

The XRD of the BD surfaces of the samples after 28 days of immersion in DPBS are presented in Figure 11. The spectra showed a strong peak of Mg3(PO4)2 for the Extr samples. Mg3(PO4)2 peaks were also present for the AB samples, while Mg(OH)2 peaks are mainly present for the HIP samples. MgO is also expected to be present to some extent, but MgO peaks overlap with those of hcp-Mg (Rettig and Virtanen, 2009). These results are in line with the results obtained from EDS, presented in Figure 12, with the cross sections of both the AB and the Extr material showing a surface layer rich in P and O for the AB material, corresponding to Mg3(PO4)2. The AB and the Extr material also have some Ca and Na present on the surface. Ca3(PO4)2 is also a common constituent forming on magnesium surfaces in Ca2+-containing corrosion mediums (Rettig and Virtanen, 2009). However, there is a morphological difference between the surface layers. The AB material exhibits a double layer structure, with one uneven layer measuring from a few µm and upwards, over a cracked surface where the beginning of the attack of the bulk material underneath can be seen. The cracked oxide layer is commonly observed in Mg alloys and their occurrence has been ascribed to the deformation of the metallic layer. This deformation is due to a combined effect from the volume expansion of the corrosion layer, the mismatch in the crystal structure between the cubic MgO and the HCP metallic Mg matrix, and the embrittlement of the metallic layer due to an uptake of hydrogen (Xu et al., 2015; Esmaily et al., 2017). For the HIP material, an uneven layer of corrosion products is visible, with a thickness in the order of 100 µm. Here the corrosion is so severe that the cracked surface morphology is not observed. EDS mapping reveals a composition consisting mainly of O and Mg, thereby suggesting a strong presence of Mg(OH)2, with some P at the surface. The Extr material, on the other hand, shows a 10–20 µm denser, and more even layer, with few cracks in it, and with the material just below this layer virtually intact. A more homogeneous microstructure has previously been proven beneficial during the initial stages of degradation, as it allows for a more even and dense formation of oxide layer (Mraied et al., 2019). Moreover, the solubility of Mg3(PO4)2 and Ca3(PO4)2 is many times lower in aqueous solutions than that of Mg(OH)2 (Aylward and Findlay, 2007). This could also explain the semi passivating behavior exhibited by the Extr material in the PDP curves.

Kalb et al. (Kalb et al., 2012) described the mechanism behind the degradation of Mg alloys, with the corrosion starting with the reduction of water at the cathodic centers under the release of Mg2+, OH-, and H2 gas. As the pH shifts upwards due to the release of OH−, Mg(OH)2 becomes stable, and precipitates at the surface of the samples. They also describe how the Mg(OH)2 converts into Mg3(PO4)2 and Ca3(PO4)2 as Ca2+ and PO43- ions diffuse into the Mg(OH)2 layer. However, as the degradation rate of the HIP material is high, it could be that the conversion of Mg(OH)2 into more stable compounds is not fast enough. The result is a porous, inhomogeneous and a relatively soluble oxide/hydroxide layer that is not protective. A similar behavior can be observed for the AB material, but as the initial degradation rate is slightly slower, part of the Mg(OH)2 has time to convert into the more stable corrosion products Mg3(PO4)2 and Ca3(PO4)2. Thus, this would mean that an initially faster corrosion rate will have a negative of effect on the stability of the corroded surfaces over time.

Thus, the results show that the HIP material has the highest degradation rate, followed by the AB material and finally the Extr material, which means that the study failed to improve the corrosion properties in relation to previous studies. The reason is ascribed to the distribution of the alloying elements and the size of the secondary phases. The larger precipitates in the HIP material increase micro galvanic corrosion, which also hinders any passive oxide layer to form. The key role of the precipitates is also a possible reason for the contradictory results of some of the long-term tests (Figure 8), where the more closely packed TD surfaces tended to give higher corrosion rates. The importance of the secondary phases for the corrosion behavior of magnesium alloys processed by PBF-LB have been established for many other alloying systems. This includes the commercial Mg-Al-Zn alloys, where there are many studies confirming the detrimental effect of the Mg-Al intermetallics on the corrosion properties (Shuai et al., 2018; Gao et al., 2021). The electrochemical activity of secondary phases is also one of the main challenges of the new alloying systems being developed for biodegradable metal implants, including the Mg-Zn-Ca alloys (Yao et al., 2021)

Another important factor that might also have affected the corrosion results is the difference in grain size distribution. A smaller average grain size and more homogenous grain size distribution has previously been proven to be beneficial for the corrosion properties of Mg-RE alloys, affecting both the distribution of secondary phases, as well as the homogeneity of the surface oxide layers (Liu et al., 2014; Liu G et al., 2022). This could thus be a contributing factor to the better corrosion property of the Extr material, as well as the better corrosion properties of the AB material in comparison to the HIP material. Moreover, this could also be the reason for the higher corrosion rates in TD direction.

It may be noted that the experiment was carried out in a static environment, and that the corrosion media was not exchanged during the 28 days of immersion. As this will affect the corrosion behavior of the material, future work should the include investigation of the corrosion behavior of the material in non-static conditions. Moreover, the number of samples available was limited as there was no possibility to produce more samples in the PBF-LB system applied. A change of PBF-LB system also entails a change in a number of process parameters such as laser spot size and argon gas flow, thus it is not possible to reproduce the samples in other systems available. The large variations in set process parameters, such as laser spot size, between different PBF-LB systems further highlights the need of an enhanced understanding of the influence of the process on the microstructure and material properties. As previously mentioned, future work is suggested to first focus on the influence of individual PBF-LB process parameters on the microstructure and the amount of evaporated Mg in order to clarify their relationship. A particular focus should be on the resulting amount and distribution of secondary phases and its overall effect on the corrosion behavior of the material. Other effects such as the influence of the PBF-LB process on the stress state of the material should also be clarified, as this could also have an adverse effect on the corrosion properties. This is important as this will expand the knowledge regarding which microstructure to aim for future process parameter development and optimization.

Finally, when the relationship among process parameters, microstructure and corrosion behavior has been clarified, further development of the Mg alloys should seek to better adapt alloy concentration to the PBF-LB process in order to compensate for the possible negative effects, such as loss of Mg during processing, and the decreasing amount of Y in solid solution due to the oxide formation. A closed value chain from powder production to the processing of the powder using PBF-LB, keeping the material under inert atmosphere, could also minimize the oxide layer to be formed on the powder, and thus the depletion of Y due to this effect. However, this would also enhance the reactivity of the powder, and thus the hazards working with it. The effect of the concentration of alloying elements on the formation of Mg-RE intermetallic compounds should also be investigated. Even though heat treatments have been ineffective thus far at improving corrosion resistance, they have not been completely optimized for PBF-LB material. This is also the case for the HIP parameters applied in this study. Future HIP experiments on PBF-LB-processed Mg-Y-Nd-Zr alloy should also explore lower temperatures and faster cooling. Moreover, once corrosion properties have been improved, the influence of texture on corrosion rates should be revisited.

## Conclusion

A typical microstructure for a Mg-Y-Nd-Zr alloy processed by PBF-LB as well as that for Mg-Y-Nd-Zr alloy processed by powder extrusion was studied. After HIP of the PBF-LB samples, a growth in secondary phases along with a larger grain size and a stronger texture was observed. While the surfaces of the AB samples showed a higher corrosion current density than that of the HIP samples, the corrosion resistance as measured by hydrogen evolution and mass change of the AB material was lower compared to the HIP material. The higher corrosion rate of the HIP samples was also confirmed by visual inspection, and BSE-SEM images of the sample cross sections. The reason for the higher degradation rates of the HIP material was attributed to the growth of secondary phases occurring during HIP, which leads to increased micro galvanic corrosion, as well as the larger grain size. To a certain extent, this indicates that the size and distribution of the secondary phases is more important than having a fully dense material. The Extr material exhibited the lowest corrosion rate, due to a higher amount of dissolved alloying elements in the matrix and less intermetallic particles and a more homogenous grain size distribution. The stability of the surface of the Extr material was further enhanced by the formation of a passivating layer of Mg3(PO4)2 and Ca3(PO4)2. Moreover, even though a stronger texture was observed along the BD, there was no significant difference in corrosion rate over time between the BD and TD surfaces. This suggests that the size and distribution of secondary phases are more critical to the corrosion resistance than the differences in electrochemical activity attributed to crystal orientation. However, further clarification of the importance of the texture as well as grain size is needed.

## Data Availability

The original contributions presented in the study are included in the article/Supplementary Material, further inquiries can be directed to the corresponding author.
